# Paraclostridium benzoelyticum Bacterium-Mediated Zinc Oxide Nanoparticles and Their In Vivo Multiple Biological Applications

**DOI:** 10.1155/2022/5994033

**Published:** 2022-05-05

**Authors:** Shah Faisal, Abdullah, Muhammad Rizwan, Riaz Ullah, Amal Alotaibi, Aishma Khattak, Nadia Bibi, Muhammad Idrees

**Affiliations:** ^1^Department of Health and Biological Sciences, Abasyn University, Peshawar 25000 KPK, Pakistan; ^2^Institute of Biotechnology and Microbiology, Bacha Khan University, Charsadda, 24460 KPK, Pakistan; ^3^Department of Microbiology, Abdul Wali Khan University, Mardan, 23200 KPK, Pakistan; ^4^Center for Biotechnology and Microbiology University of Swat, KPK, Pakistan; ^5^Department of Pharmacognosy (Medicinal Aromatic and Poisonous Plants Research Center), College of Pharmacy King Saud University, Riyadh, Saudi Arabia; ^6^Department of Basic Science, College of Medicine, Princess Nourah Bint Abdulrahman University, P. O. Box 84428, Riyadh 11671, Saudi Arabia; ^7^Department of Bioinformatics, Shaheed Benazir Bhutto Women University, Peshawar, KPK, Pakistan; ^8^Department of Microbiology, Shaheed Benazir Bhutto Women University, Peshawar, KPK, Pakistan; ^9^Department of Biotechnology, University of Swabi, KPK, Pakistan

## Abstract

We presented a low-cost, eco-friendly, and efficient bacterium-mediated synthesis of zinc oxide nanoparticles (ZnO-NPs) utilizing Paraclostridium benzoelyticum strain 5610 as a capping and reducing agent. Scanning electron microscopy, X-ray diffraction, Fourier transform infrared spectroscopy, energy-dispersive X-ray, and UV-vis spectroscopy were used to physiochemically characterize the biosynthesized ZnO-NPs. A major narrow peak at 441 nm was observed using UV-visible spectroscopy, verifying the presence of nanoparticles. According to SEM and TEM studies, the average dimensions of ZnO-NPs was 50 nm. The crystal size of 48.22 nm was determined by XRD analysis. FTIR analysis confirmed the presence of various reducing metabolites on the surface of ZnO-NPs. The synthesized nanoparticles were investigated for biological activity against Helicobacter suis, Helicobacter bizzozeronii, Helicobacter felis, and Helicobacter salomonis. Helicobacter suis was the most vulnerable strain, with an inhibitory zone of 19.53 ± 0.62 mm at 5 mg/mL dosage. The anti-inflammatory and the findings of the rat paw edema experiments revealed that the bacterium-mediated ZnO-NPs had a strong inhibitory action. In the arthritis model, the solution of ZnO-NPs showed 87.62 ± 0.12% inhibitory effect of edema after 21 days when linked with that of the standard drug. In the antidiabetic assay, ZnO-NPs sharply reduced glucose level in STZ-induced diabetic mice. In this study, the particle biocompatibility by human red blood cells was also determined. Keeping in view the biological importance of ZnO-NPs, we may readily get the conclusion that Paraclostridium benzoelyticum strain 5610-mediated ZnO-NPs will be a prospective antidiabetic, antibacterial, antiarthritic, and anti-inflammatory agent in vivo experimental models and can be used as a potent antidiabetic drug.

## 1. Introduction

When it comes to nanotechnology, it is a fascinating and well-established topic that has yielded a slew of objects with distinct functions and uses in a wide variety of sectors. Several technologies (biosynthetic, physical, and chemical) are available for the production of nanomaterials, and they have a diverse spectrum of characteristics and applications. Nanomaterials are materials having at least one dimension ranging from 10 to 100 nanometers on the nanoscale (on the size of an atom) [1, 2]. More than 50,000 publications on nanotechnology are published every year, while the European Patent Office gets more than 2,500 patent applications [1]. Human living standards have also been raised as a result of nanotechnology's success in addressing a variety of daily challenges such as energy security, climate change, the beauty, textile, and health industries and the treatment of deadly diseases such as cancer and Alzheimer's disease [3]. Metal oxide nanoparticles (NPs) have been the subject of much research over the last decade, owing to their wide range of applications in a variety of technical sectors [4]. Due to their particular chemical and physical features, zinc oxide nanoparticles (ZnO-NPs), which are the most important metal oxide nanoparticles, are widely used in a wide range of industries [5, 6]. ZnO nanoparticles (ZnO-NPs) were first used in the rubber industry to boost the toughness and intensity of high polymers while also giving antiaging properties [7, 8]. The zinc oxide (ZnO) is commonly utilized in personal care products such as cosmetics and sunscreens because of its outstanding UV absorption properties [9]. ZnO nanoparticles also have exceptional antibacterial, antimicrobial, and UV-blocking properties, among other properties. As a consequence, finished textiles incorporating ZnO-NPs in the textile industry have the desirable attributes of being antibacterial, deodorant, and resistant to UV and visible light, among other things [10]. In addition to the industries described above, zinc oxide may be found in a variety of other fields, such as concrete manufacture, photocatalysis, electronics, and electrotechnology [11, 12]. In addition to being a unique technique for transporting zinc, zinc oxide nanoparticles (ZnO-NPs) have crucial implications in the treatment of a range of disorders, including diabetes [13]. Zinc supplementation has been demonstrated to have an ameliorative impact in preclinical investigations [13, 14]. As a result, the development of a zinc-based medication for the treatment of diabetes and its complications might be promising [15]. Several studies have looked at the antidiabetic benefits of ZnO-NPs via increasing insulin receptors, insulin, and glucose-metabolizing enzyme gene expression [16]. ZnO have a variety of biological uses, including antioxidant and anti-inflammatory properties [17]. The basic material for the production of ZnO-NPs is plants or microbes. Nanoparticle creation necessitates bacteria's capacity to withstand high levels of heavy metal toxicity, and these germs produce nanoparticles under stress by converting metal ions to metal oxide [18]. Microorganisms contribute to the reduction of metal ions by releasing or producing a range of biomolecule complexes [18]. Clostridia are made of a wide range of Gram-positive, spore-forming, and anaerobic bacilli, whose taxonomic taxonomy of genera has been revised. Toxin-producing organisms may cause moderate to life-threatening illnesses, the most renowned being the genus Clostridium (Clostridium botulinum, Clostridium perfringens, and Paraclostridium benzoelyticum), the genus Paeniclostridium (Paeniclostridium sordellii), and the genus Clostridioides (Clostridioides difficile) [19].

In light of this, we sought to produce ZnO-NPs from Paraclostridium benzoelyticum strain 5610, and UV spectroscopy, XRD, SEM, EDX, and FTIR were used to analyze the bioinspired produced ZnO-NPs. Furthermore, antidiabetic, antiarthritis, nephrotoxicity, leukocyte migration assay, biocompatibility, and antibacterial tests were performed on the produced nanoparticles. The best of our information is that this is the first publication report that successfully synthesizes ZnO-NPs utilizing Paraclostridium benzoelyticum.

## 2. Materials and Method

### 2.1. Synthesis of ZnO-NPs using Paraclostridium benzoelyticum

ZnO-NPs were made using a previously described method, which included new aspects [20]. Paraclostridium benzoelyticum strain 5610 was isolated from the side of the road and identified (GenBank accession number MT510437.1). Paraclostridium benzoelyticum strain 5610 was inoculated in nutrient broth (NB) and incubated for 24 hours before diluting for at least four times with fresh nutrient broth to a final volume of 100 mL and incubated for another 24 hours. Furthermore, 0.1 M zinc nitrate was given through drops to this overnight developed culture (OD > 1 at 600 nm) and then heated at 80°C for 10 minutes after a white precipitate emerged at the bottom of the flask and then incubated for another 24 hours. Several centrifugations at 14,000 rpm for 10 minutes were used to purify ZnO-NPs and then dried at 120°C. For all subsequent characterization and biological research, the dried pellet was kept.

### 2.2. Physicochemical and Morphological Characterization

UV-vis spectroscopy, Fourier transform infrared spectroscopy, scanning electron microscopy, X-ray diffraction, and transmission electron microscopy testing were used to examine the chemical, vibrational, structural, and structural properties of biosynthesized ZnO-NPs [21]. The crystallinity of a material was determined using a model D8 Advance XRD (Germany) with the size of the scanning step of 0.03°/s in a temperature range of 2 v (10°-80°). Zn K radiation was required to gather diffraction data (wavelength 1.5406, generator voltage 40 kV, and tube current 30 mA). Scherer's equation was used to compute the crystallite size, as shown below [22].

(1) $$\begin{array}{c} D=k\frac{\lambda }{\beta \text{Cos}\theta }. \end{array}$$

X-rays have a wavelength of 1.5421 Å and a shape factor of 0.94, a value of the Bragg angle.

The integrity of synthesized NPs was tested using EDX analysis. In the FTIR analyses discovered as a result of capping and reducing agents in the extract, functional groups on NPs have been formed. To visually investigate particle scattering stability, this experiment was done out in clear water with various pH values. Scanning electron microscop(SEM) was utilized to assess the size and morphology of biogenic ZnO-NPs [23].

### 2.3. Antibacterial Action of ZnO-NPs against Bacterial Strains of H. pylori

The antimicrobial activity of ZnO-NPs was tested in vitro with minimal changes making use of the agar well diffusion method [24]. To begin, the seeding density of the bacterial culture (1 × 10^−6^ CFU/mL) was adjusted to get the most efficient seeding density. 50 liters of newly cultivated microorganisms was used to make the nutrient agar lawn. In each well, a 10-microliter test sample was inserted. After that, in the same way, the seeded plates were labelled. On the seeded plates, the names were spelled out as well. Antibiotic ampicillin was utilized as a positive control, whereas DMSO was employed as a negative control. The inhibitory zone was evaluated in all test wells containing samples and controls following a 24-hour period of incubation at 37°C. A Vernier caliper's mm gauge was used to measure the thickness of the zones.

### 2.4. Induced Paw Edema in Rats by Carrageenan

The test group of rats developed edema in their right hind paws after being injected on the plantar side with 0.2 mL of 1 percent (*w*/*v*) carrageenan (Sigma-Aldrich; St. Louis, USA), with a volume of 0.2 mL [25]. The paw diameter was measured before and after the carrageenan injection. After the carrageenan injection, the paw diameter was measured every hour for up to 5 hours and then every 24 and 48 hours. The rats were divided into three groups, each with six rats. The first group was given normal saline (3 mL/kg body weight), whereas the second was given diclofenac (Troge; Germany) (100 mg/kg body weight p.o.) as a traditional anti-inflammatory medicine. The animals in the third group were given NPs (400 mg/kg body weight p.o.) One hour before, they were given carrageenan, as well as a pretreatment. We calculated the proportion of edema inhibition using this method: %inhibition of edema = mean edema increase in control group–mean edema increase in treated group × 100. In the control group, the mean edema increased.

### 2.5. Adjuvant-Induced Chronic Arthritis

The rat's left footpad was injected with complete Freund adjuvant, which led the animal to develop experimental arthritis (Difco Laboratories, CFA, MI, Detroit, USA). After the CFA challenge, the rats were given NPs (500 mg/kg body weight p.o.) daily for 21 days, same as the test groups. To be specific, the third group received diclofenac (10 mg/kg body weight p.o.); the control group, on the other hand, was given saline solution (3 mL/kg body weight p.o.).

### 2.6. Leukocyte Migration Assay

As previously reported, air pouches in the dorsal subcutaneous (20 mL sterile air) were generated in four groups of rats. Three groups received carrageen (1%), and one group received 0.9 percent NaCl on the third day after the cavity was created. After the air pouches therapy, the rats in the test groups were given a 4-day treatment with ZnO-NPs (500 mg/kg body weight p.o.). Those who received it (3 mL/kg body weight) in the control group were given saline. Individuals who required diclofenac (100 mg/kg body weight) were placed in the third group. By injecting five milliliters of ice-cold NaCl 0.9 percent into the cavity and collecting it, the leukocyte count was determined [26].

### 2.7. Experimental Diabetes Induction

Trial rats were administered STZ (45 mg/kg body weight) intraperitoneally (i.p.) to progress diabetes [27]. Blood was obtained for 5 days after mice were injected with STZ dissolved in newly made 0.01 M sodium citrate buffer (pH = 4.5). To lessen the threat of hypoglycemia following STZ injection, the animals can drink a glucose solution (5 percent *w*/*v*) overnight. In the control group, the mice were simply fed the agents (citrate buffer). After five days of treatment, STZ-treated mice were permitted to return to their regular habitat. Mice were put on a diet for two weeks after fasting developed diabetes and had high level of blood glucose greater than 11.1 mmol/L. The STZ-treated mice were tethered for 12 hours, and the blood samples from their tail veins were collected to measure blood glucose levels. Diabetic mice were diagnosed as having blood glucose levels more than 11.1 mmol/L after fasting and were selected for additional testing.

### 2.8. Animal Grouping

The mice with diabetic were placed into four groups, each with five animals, for the test of antidiabetic activity. G and I mice were separated into two groups, each receiving only distilled water. The results of these tests may be classified into four groups. Mice with diabetes was administer subcutaneously with 0.4 units of insulin (per body weight of 50mgin the usual treatment group [G-II (ZnO-NPs treated), G-III (ZnO-NPs and insulin-treated), and G-IV] (s.c.). The dosages were given to each group over the course of 14 days. A total of twenty mice were chosen and distributed into four groups. The study was then carried out using a well-established methodology [27]. Three distinct studies looked at the effects of different mouse groups (G-II, G-I, and G-IV) on mice. An OGTT is an oral glucose tolerance test that can be interpreted as follows: six hours after fasting, mice were given 2 g of glucose per kilogram of body weight. Following that, they were given glucose by mouth before receiving ZnO-NPs. After being injected into the circulation, blood glucose levels were measured at 0, 15, 30, 60, and 90 minutes. The second and third groups received ZnO-NPs (8 mg/kg b.w., oral), ZnO-NPs (14 mg/kg b.w., oral), and glibenclamide (10 mg/kg b.w., oral), respectively [28].

#### 2.8.1. Biochemical Determination

The glucose oxidase technique of the “ACCU-CHEK Active” kit was used to determine glucose level in blood. The animals' blood glucose levels were checked before they started the experiments. Until diabetes was diagnostic, fasting blood glucose levels were checked often.

#### 2.8.2. Protocol for Sampling

Blood was collected from the tail veins of all the animals in the study for 2–3 mL. The blood sugar levels were monitored using an ACCU-CHEK-active blood glucose meter.

### 2.9. Biocompatibility Assay

The biocompatibility of biogenic ZnO-NPs was demonstrated using fresh human red blood cells (hRBCs). With their permission, healthy people's 1 mL blood samples were obtained in EDTA tubes. RBCs were isolated by centrifugation after blood samples were collected. Following centrifugation, a supernatant and pellet were generated; the supernatant was discarded, and the pellet was recovered after three PBS washes. PBS-erythrocyte suspension is made by mixing 200 L of RBCs with 9.8 mL of PBS (pH: 7.2). In Eppendorf tubes, the erythrocyte suspension and green-produced ZnO-NPs were mixed together. The erythrocyte suspension and NPs were then incubated in Eppendorf tubes for 1 hour at 35°C. 200 L of the supernatant was transferred to a 96-well plate after centrifuging the reaction mixture at 12,000 rpm for 10 minutes, and haemoglobin release absorption spectra at 450 nm were recorded. To calculate % haemolysis, use the following formula:

(2) $$\begin{array}{c} \left(\%\right)\text{ Haemolysis}=\left(\frac{\text{sample Ab}-\text{negative control Ab}}{\text{Positive control Ab}-\text{Negative control Ab}}\right)\times 100, \end{array}$$

where Ab denotes the reported absorbance of the samples.

### 2.10. Nephrotoxic Effect of ZnO-NPs

The National Institutes of Health gave albino mice for the research. For 7 days prior to the studies, the animals were maintained in a temperature adjust environment with 12 hours of light and 12 hours of darkness (1 week). The animals were divided into four groups, each with six animals. The first and second groups got an intraperitoneal injection of normal saline for 7 and 14 days, respectively. For 7 and 14 days, the third and fourth groups received 0.1 ml of 150 mgkg^−1^ ZnO-NPs, respectively. Mice were given unrestricted access to commercial pellets and fresh tap water regularly. Seven and fourteen days after treatment, male mice were killed by cervical dislocation. The kidneys were prepared for histological examination. First, a kidney specimen was preserved overnight in buffered neutral formaldehyde at room temperature. After that, the samples were dehydrated in a succession of ethyl alcohols, treated with xylene, and finally embedded in paraffin wax. 5 m thick histological slices were cut using a rotary microtome, and chosen slides were stained with hematoxylin and eosin. After that, an Olympus digital camera and optical light microscopy were used to examine the slices (Sony, Japan). ImageJ was utilized to do semiquantitative measures to identify structural changes in the kidney (Biometric).

## 3. Results

### 3.1. ZnO-NP Synthesis

**Figure 1:**
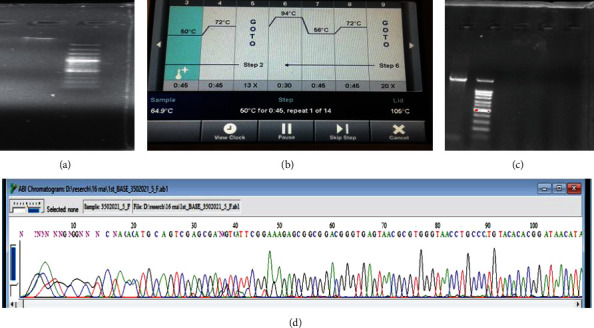
Molecular identification of *Paraclostridium benzoelyticum* strain 5610: (a) Gel image of DNA), (b) PCR image, (c) gel image of PCR product, (d) Sanger sequencing peaks.

**Figure 2:**
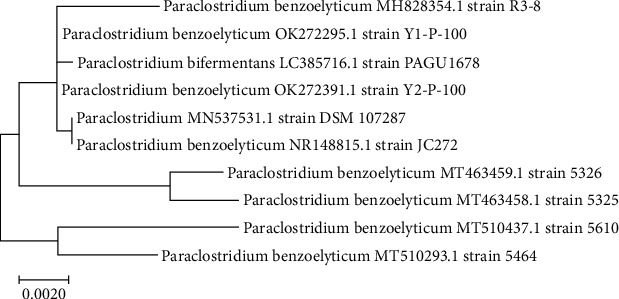
Phylogenetic study of the benzoelyticum strain 5610 16S rDNA sequence employed in the green production of ZnO-NPs.

**Figure 3:**
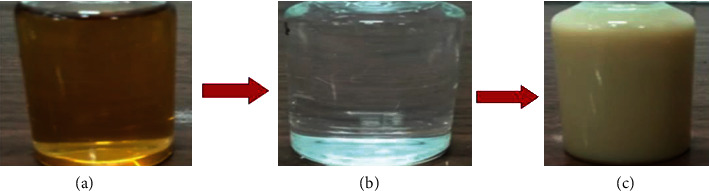
The entire process of making ZnO-NPs is depicted in this schematic picture. (a) Paraclostridium benzoelyticum strain 5610 filtrate. (b) Zinc nitrate solution (0.1 M). (c) Zinc nitrate and Paraclostridium benzoelyticum strain 5610 extract mixed together.

The 16SrRNA gene was used to conduct the molecular identification. The genomic DNA extracted from the isolates was amplified using 16S rRNA primers, as described in Materials and Method of this report. In order to accomplish molecular identification, we must first extract the DNA and then run a PCR on it. For all of the isolates, the size of the amplified product of the PCR was roughly 1000 bp. This is seen in Figure 1. Following the sequencing process, a partial 16S gene was acquired. The partial 16S rRNA sequence was retrieved in FASTA format and submitted to a BLAST search against the GenBank database. Different findings demonstrate that the phylogenetic study of these strains improved their performance, although MEGA-7 was by far the most significant. Based on homology and matches with previously known bacterial rRNA sequences (Figure 2), the bacteria were identified as Paraclostridium benzoelyticum strain 5610, and the sequence was submitted to the NCBI GenBank database (accession number MT510437.1). Nanosized minerals and other metallic nanoparticles are produced by a variety of microbes. Because the bacteria control the nanoparticles size, shape, and content, they have qualities similar to chemically produced materials. However, the microorganisms used in the production process greatly impact the nanoparticle's physical qualities such as shape, size, and crystallinity. Therefore, the existence of a white precipitate, which was a sign of the bacteria's reaction mixture with the precursor salt, zinc sulphate, could be easily monitored to ensure that bacterium-modified ZnO-NPs were successfully created. When the 0.1 M zinc nitrate solution was introduced to the bacterial culture drop by drop, a white precipitate was formed, and the reaction mixture color changed from neutral to whitish, indicating the synthesis of ZnO-NPs, as shown in Figure 3. After 24 hours, a whitish precipitate was formed, indicating that zinc ions had been reduced and ZnO-NPs had formed.

### 3.2. Ultraviolet-Visible Spectroscopy of ZnO-NPs

**Figure 4:**
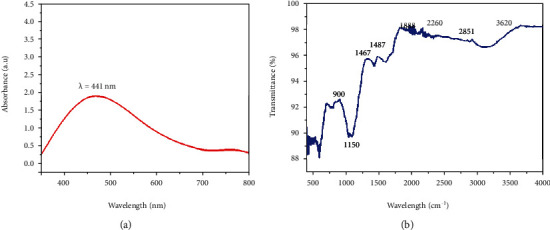
(a) UV visible spectrum and (b) typical FTIR spectra of Paraclostridium benzoelyticum strain 5610-synthesized ZnO-NPs.

UV-visible spectroscopy in the 300 nm to 800 nm range shows that the dark brown color is a sign of nanoparticle formation. An analysis of zinc nanoparticle absorption reveals a definite peak at 441 nm at 2.25 au, proving that they were made.  Figure 4(a) illustrates this. There is just one SPR peak seen in metallic nanoparticles, found in zinc nanoparticles.

### 3.3. Fourier Transform Infrared Spectroscopy of ZnO-NPs

Researchers utilized Fourier transform infrared spectroscopy to look for different functional groups in the bacterial molecules that may have had a role in capping or reducing metal ions in the biosynthesized nanoparticles. Various peaks were found in the spectral range of alkanes, alcohols, carboxylic acids, and conjugated acid halides, as shown in Figure 4(b). These peaks correspond to the stretching of C-C bonds and C-O bonds, bending of C-H bonds, and stretching of C=O bonds in carboxylic acids and conjugated acid halides. In addition, we found that reducing particles to their smallest size also causes particle.

### 3.4. X-Ray Diffractive Analysis of ZnO-NPs

**Figure 5:**
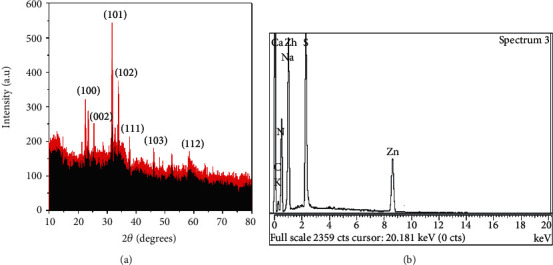
(a) Typical XRD pattern and (b) EDX spectrograph of Paraclostridium benzoelyticum strain 5610-synthesized ZnO-NPs.

X-ray diffractive analysis was used to explore ZnO-NP crystalline structure. As shown in Figure 5(a), the prominent peaks correspond to lattice plans of (100), (002), (101), (102), (110), (103), and (112) correspondingly at Braggs angles of 21.7°, 26.11°, 33.16°, 35.11°, 39.61°, 48.88°, and 56.31°. According to the XRD findings, the biosynthesized nanoparticles exhibit a face-centered cubic (fcc) crystal structure. The peaks identified were used to allocate lattice planes, when utilizing the Joint Committee on Powder Diffraction Standards (JCPDS) as a guide. Also, the nanoparticles generated had an average diameter of around 48.22 nm. The Debye-Scherer equation was used to figure out the nanoparticle's size.

### 3.5. EDX Analysis

Biosynthesized ZnO-NPs underwent energy-dispersive X-ray examination to determine their elemental composition. This zinc EDX spectrum shows two separate intense zinc peaks at 1.2 and 8.4 keV. Sodium (Na), nitrogen (N), oxygen (O), sulphur (S), and carbon (C) showed additional peaks at various kilo electron volts (keV) is shown in Figure 5(b). Additionally, the bacterial extract converted zinc nitrate to nanoparticles, which resulted in the additional EDX peaks.

### 3.6. Analysis of ZnO-NPs by SEM

**Figure 6:**
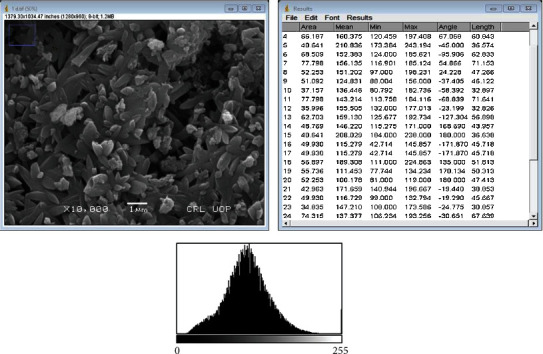
SEM and ImageJ analysis and size distribution histogram of ZnO-NPs.

Nanoparticle size and form are seen via SEM analyses. SEM used to determine the morphology of ZnO-NPs. In addition, SEM was utilized to examine the ZnO-NPs' overall appearance in the present research.  Figure 6 shows the SEM micrograph of the produced ZnO-NPs, which verified their spherical and rectangular shape and particle size. The SEM at various magnifications indicated the presence of spherical nanoparticles with an average diameter of 50 nm, thus demonstrating their existence. Furthermore, the produced ZnO-NPs had a hexagonal shape, agreeing with our findings.

### 3.7. Antibacterial Assay

**Figure 7:**
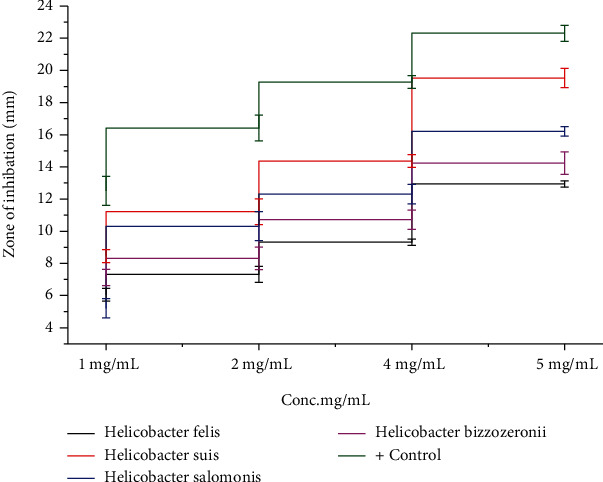
Antibacterial properties of ZnO-NPs produced at various concentrations.

**Figure 8:**
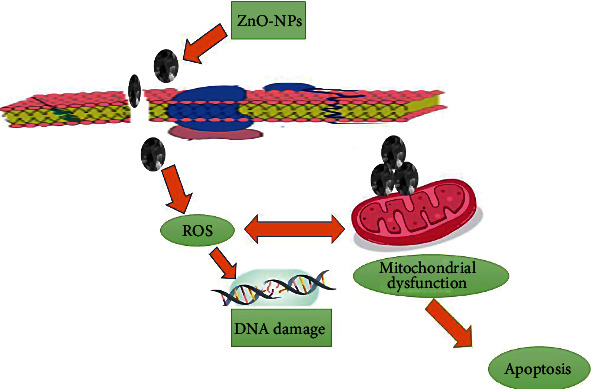
Mechanistic approach of Sno2 NPs for cytotoxicity.

The antibacterial potential of ZnO nanoparticles was tested against four pathogens: Helicobacter felis, H. suis, H. salomonis, and H. bizzozeronii. The generation of reactive oxygen species (ROS) as a consequence of light and the breakdown of metal oxide nanoparticles plus the electrostatic correspondence of nanoparticles with microbe cell walls have all been postulated as modes of action for metal oxide nanoparticles against bacteria. The aggregation of ZnO-NPs mostly on the exterior of the bacterial membrane could be a reason for their antibacterial activity. ZnO-NPs create reactive oxygen species (ROS), which consort with the bacterial membrane, causing membrane permeability and cell death. Metal nanoparticles and their oxides are one of the most promising strategies for treating bacteria antibiotic resistance. The disc diffusion approach, which uses ZnO-NPs, was used to demonstrate antibacterial activity in Figure 7. The release of Zn4+ damages mitochondria as well as DNA of Cell and is capable of inhibiting the Cell key enzymes ultimately triggering the death of cell as seen in Figure 8. Antibiotic resistance in bacterial strains is one of the most important problems in world health care. Four pathogenic bacteria strains, Helicobacter felis, H. suis, H. salomonis, and H. bizzozeronii, were confirmed in the presence of NPs at concentrations extending from 5 mg/mL to 0.5 mg/mL. The positive control in these experiments were antibiotic ampicillin. The bactericidal activity of a bacterial strain tested was greatest when ZnO-NPs were introduced to a dosage of 5 mg/mL to the medium. At greater concentrations, ZnO-NPs were shown to be bactericidal, but at lower quantities, they were found to be bacteriostatic. Helicobacter felis, Helicobacter salomonis, Helicobacter suis, and Helicobacter bizzozeronii all had good inhibitory zones against 5 mg/mL, with 16.21 ± 0.35, 12.94 ± 0.22, 19.53 ± 0.62, and 14.23 ± 0.72, respectively. Because of their distinct morphologies, diameters, and biocompatibility, biofabricated ZnO-NPs have gotten a lot of interest as an antibacterial treatment.

### 3.8. Anti-inflammatory Assay In Vivo

#### 3.8.1. Reduction of Carrageenan-Induced Paw Edema in Rats

**Figure 9:**
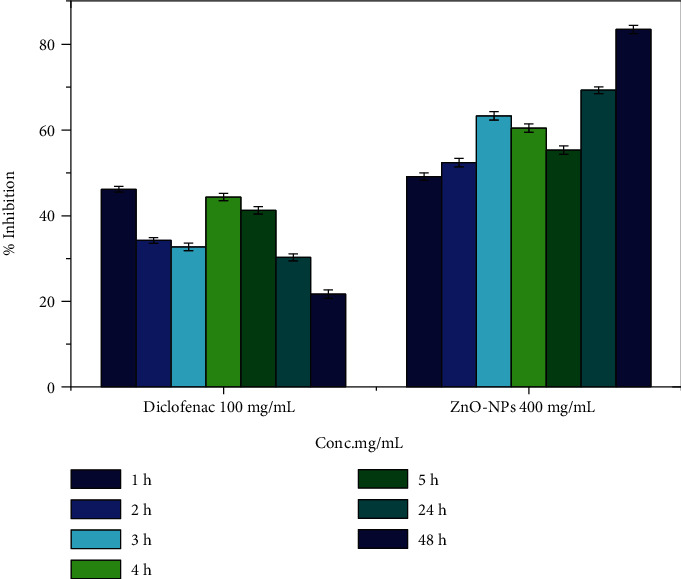
The effect of ZnO-NPs on carrageenan-induced edema in rats' hind paws.

**Figure 10:**
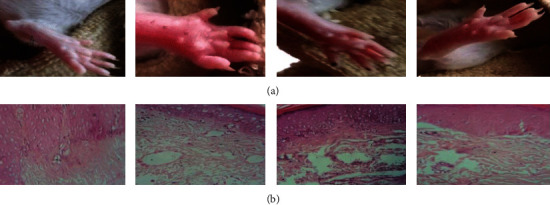
Carrageenan-induced paw edema in rats is reduced. a) A picture of Paw. b) Paw edema histopathology.

**Figure 11:**
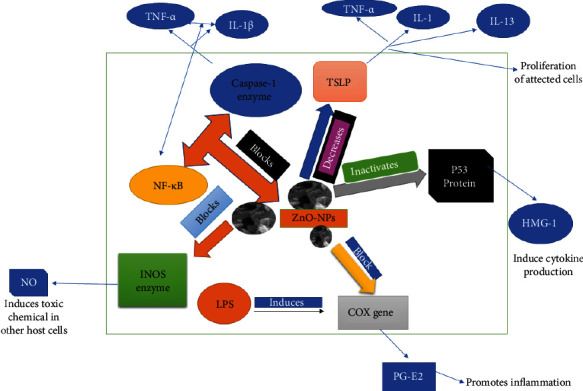
Mechanistic approach of ZnO-NPs for anti-inflammatory effects.

Proinflammatory cytokines such as interleukin-1, interleukin-1 beta, and tumor necrosis factor-alpha are involved in the upregulation of inflammatory responses, and their production results in inflammation. Mast cells are known to differentiate and proliferate when exposed to certain substances. Nuclear factor kappa B (NF-B) is a transcription factor that activates the expression of particular genes in order to maintain the proliferation of affected cells, hence intensifying the inflammatory response. IL-1-converting enzymes, such as the caspase-1 enzyme, are responsible for the conversion of inactive cytokines, such as pro-IL-1 and pro-IL-18, into their active counterparts. By producing numerous inflammatory mediators such as histamines, chemokines, leukotrienes, and cytokines, mast cells are recognized to play significant immunoregulatory functions in immunological diseases. They also cause allergic inflammatory reactions by promoting the production of IgE by B-lymphocytes in the body. ZnO-NP inhibits the caspase-1 enzyme in activated mast cells, as well as the nuclear factor-B (NF-B). It has been proven that ZnO nanoparticles inhibit LPS-induced nuclear factor-B (NF-B) by upregulating A20, which is a negative regulator of nuclear factor-B in RAW 264.7 macrophages. Also shown to limit the nuclear translocation of NF-B and p65 (an NF-B family member) that was increased by LPS, as well as to decrease the cytosolic degradation of IB, a cellular protein that suppresses the transcription of the NF-B transcription factor TNF- and interleukin-1 (IL-1) synthesis is both proinflammatory cytokines, and as a result, this inhibits their production. In addition, zinc oxide nanoparticles (ZnO-NPs) inhibit the generation of malondialdehyde (MDA). MDA is a well-established measure of oxidative stress, and it has been hypothesized that ZnO-NP may also aid in the reduction of oxidative stress. They also have the additional effect of reducing myeloid peroxidase levels, which are mostly present in neutrophils, hence decreasing neutrophil activity. ZnO-NPs are an effective healing agent for reducing inflammation in acute circumstances, according to the present data (Figures 9 and 10). The mechanistic approach of ZnO-NP for inflammation of the rat's paw generated by the substance carrageenan commonly employed in the pharmaceutical sector to find novel anti-inflammatory drugs is shown in Figure 11. Injection of carrageenan causes an increase in serotonin, prostaglandin, and histamine-like chemicals in the rat's paw, causing edema. In rat paw edema tests, plant-mediated ZnO-NPs demonstrated considerable inhibitory activity. After 48 hours, the ZnO-NP-treated group had 83.52 ± 0.18% less edema than the control group, which received diclofenac (100 mg/kg). The amount of white blood cells travelling to the inflammatory site had increased, according to our finding. Prostaglandins were implicated in the inflammatory process in this test model. Inflammatory mediators that enhance vascular permeability and/or mediators that increase blood flow produce edema in the paw.

#### 3.8.2. Reduction of Adjuvant Arthritis-Induced Paw Edema

**Figure 12:**
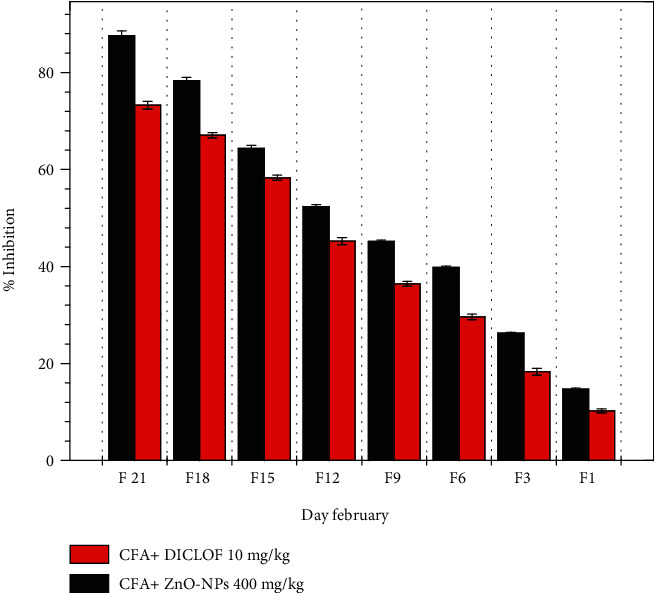
Reduction of adjuvant arthritis-induced paw edema.

**Figure 13:**
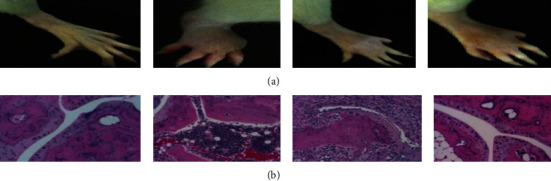
Reduction of adjuvant arthritis‑induced paw edema in rats. (a) Picture of rat knee joint. (b) Histopathology of rat knee joint tissue.

Preinjection of ZnO-NPs demonstrated that by delivering the cure for a total of 21 days, it was feasible to minimize paw edema in rats when compared to the control group (Figures 12 and 13) which show that ZnO-NPs resulted in an 87.62 ± 0.12 percent decrease in edema after 21 days. ZnO-NPs were shown to be more effective than conventional medication at reducing CFA-induced chronic inflammation in the rat knee joint in a current study. In CFA-induced monoarthritis, the ZnO-NPs tested resulted in a substantial reduction in paw volume when compared to the control. Terpenoid and phenolic chemicals have anti-inflammatory properties, which might describe the anti-inflammatory properties found in this study. According to data obtained using the leukocyte migration test, the mechanism of actions of NPs appears to include altering leukocyte migration into target organs and tissues. Both etiology research and the assessment of emerging natural treatments frequently utilize adjuvant arthritis.

#### 3.8.3. Migration of Leukocytes/Leukocyte Migration

**Figure 14:**
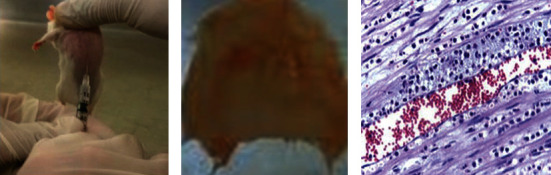
Leukocyte migration assay using ZnO-NPs.

As indicated in Figure 14, the number of leukocytes driven to the air-induced hollow following carrageenan injection alone was larger (6 × 105 cells/mL) than the number of leukocytes observed in the groups treated with diclofenac (4.7 × 10^6^ cells/mL) or treated with NPs (4.1 × 105 cells/mL).

### 3.9. In Vivo Antidiabetic Assay

**Figure 15:**
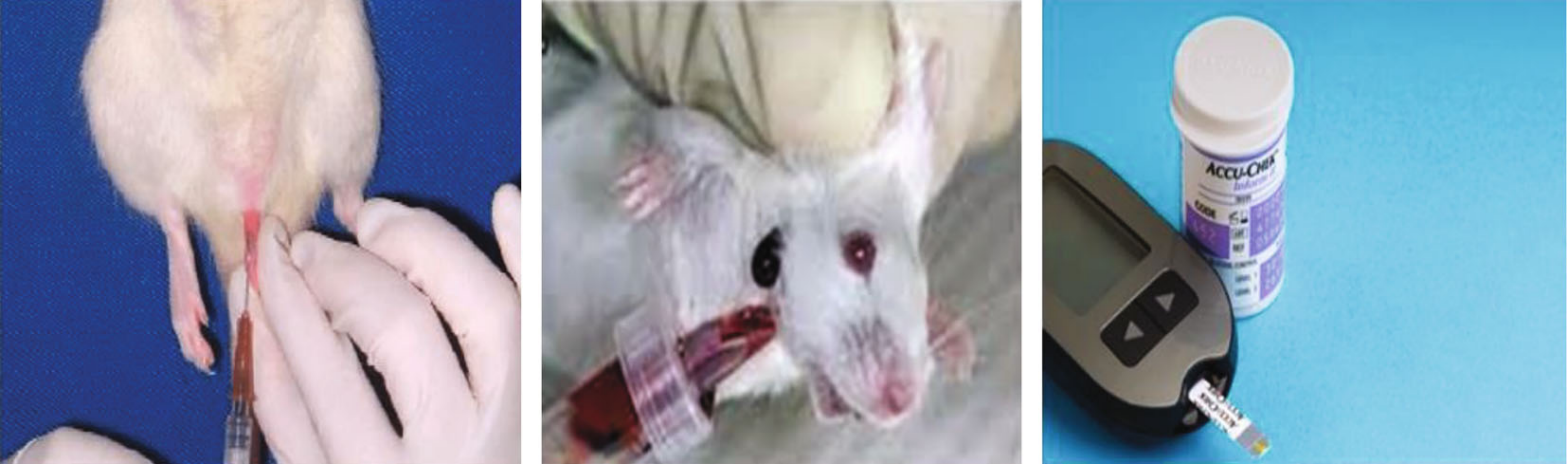
Antidiabetic assay for ZnO-NPs.

Injecting ZnO-based nanoparticles into diabetic mice resulted in significant decreases in blood glucose levels when the mice are not provided anything to eat as seen in Tables 1 and 2. Hepatic gluconeogenesis and glycogenolysis, which increase excessive glucose synthesis while limiting glucose utilized by the tissues, induce hyperglycemia. Discovered ZnO-NPs significantly reduced glucose levels in STZ-induced diabetic mice. The NPs block the active site and thus prevent substrate binding as shown in Figure 15. The collections of mice that were given a treatment with ZnO-NPs and ZnO-NPs+insulin showed 36.89 percent and 74.38 percent decreases in blood glucose levels, respectively, when compared to the control group.

### 3.10. Biocompatibility with hRBCs

**Figure 16:**
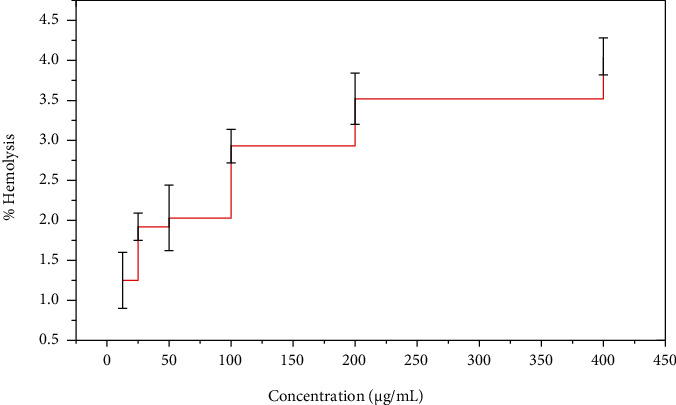
Biocompatible nature of ZnO-NPs against human red blood cells (hRBCs).

The fraction of human blood affected by high ZnO-NP concentrations (ZnO-NPs at concentrations ranging from 400 g/mL to 12.5 g/mL) has been discovered to be a valuable tool for learning about the negative effects of ZnO-NPs on human exposure. Human red blood cells were extracted from the circulatory system and evaluated in a study to see if they were biocompatible when combined with ZnO-NPs. The proportion of hemolyzed ZnO-NPs confirmed the cytotoxic effect of these produced ZnO-NPs at the greatest absorption, as shown in Figure 16, i.e., at 400 g/mL. At 12.5 g/mL, the percentage was 1.25 ± 0.35, and at 4.05 ± 0.23 g/mL, the percentage was 4.050.23. Furthermore, it was revealed that the haemolysis effectiveness of ZnO-NPs reduces when the concentration of ZnO-NPs is reduced. According to our findings, the nontoxic of ZnO-NP concentration is 12.5 g/mL, and hemolysis possibility is proportional to NP size.

### 3.11. Nephrotoxic Effect of ZnO-NPs

**Figure 17:**
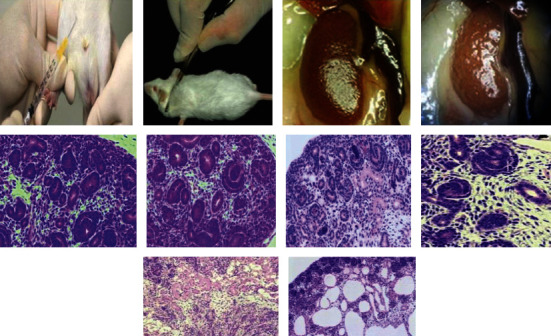
Nephrotoxic effect of ZnO-NPs in various groups.

When compared to the control group, the current findings demonstrated a change in the diameter of renal corpuscles and a reduction of glomerular diameter following 14 days of injection with 150 mg kg-1 ZnO-NPs (Table 3). Bowman's space distance increased statistically substantially (*P* 0.05) after 7 and 14 days after injection with 150 mg kg^−1^ ZnO-NPs as compared to the control group (Table 3). When compared to control groups, the diameter of renal tubules (proximal and distal tubules) increased statistically substantially (*P* 0.05) following injection with 150 mg kg^−1^ ZnO-NPs for 7 and 14 days (Table 4). In the control groups, histological evaluation of the kidney tissues showed no significant changes from the usual histological structure (Figure 17). After a 7-day intraperitoneal injection of 150 mg kg^−1^ ZnO-NPs, infiltration of inflammatory cells, sloughing and damage of the lining epithelium in renal tubules, nucleus hypertrophy foci, epithelial cell necrosis, glomerulus loss blood vessel congestion, and atrophy, and intratubular calcium deposition were all observed. After 14 days of injection, the glomerulus shrank, whereas intratubular calcium deposition increased. After 14 days, the histological abnormalities in the kidneys were more pronounced than on day 7.

## 4. Discussion

The Bio-inspired nanoparticle synthesis approach has acquired some traction in the scientific community in recent years, owing to its ease of use, nontoxic nature, quick turnaround time, low cost, and efficacy in large-scale manufacturing, among other factors. With the help of the bacteria Paraclostridium benzoelyticum strain 5610, ZnO-NPs were produced and tested for antidiabetic, antiarthritic, nephrotoxic, leukocyte migration assay, biocompatibility, and anti-H. pylori properties. A green synthesis strategy was used to conduct the tests. Different analytical methods were used to analyze the ZnO-NPs, including FTIR, UV-visible spectroscopy, XRD, EDX and SEM. According to UV-visible spectroscopy, the sample absorbed energy at 441 nm, which corresponds to a characteristic peak for ZnO-NPs. In a similar vein, [29] came to the same result. Furthermore, the absence of any further peaks in the absorption spectrum at 441 nm indicated that the nanoparticles were completely pure. After conducting multiple investigations, it was observed that ZnO-NPs exhibit a large absorption peak beneath the 450 nm wavelength, which was related to the samples' red shift at 500 and 700 degrees Celsius. They also said that when an electron gains energy via a material transition, it moves from a lower to a higher energy level [30] [31]. According to FTIR analysis of ZnO-NPs mediated by the bacteria Paraclostridium benzoelyticum strain 5610, the stretching vibration peaks of ZnO-NPs were found to be alkyl-halides, aliphatic amines, phenol, alkenes, aromatics, and aliphatic amines [32]. In a similar vein, comparable outcomes have been observed. The peaks in carboxylic acid, amino acid, and polysaccharide were shown to be caused by –C=O–, C–O–C, and C–O stretching vibrations, which were all previously thought to be caused by –C=O– stretching vibrations. This and other discoveries were published by [31], who reported a series of findings that were substantially comparable to this one. The spherical and rectangular shapes of the ZnO-NPs were validated by SEM micrographs, which were found to have particle sizes in the range of 50 nm on average, according to nanomeasurer and ImageJ measurements. More importantly, even though the size of the nanoparticles produced in this study was larger than that of previous studies, the shape of ZnO-NPs produced was monoclinic, which is compatible with our findings [32]. Changing manufacturing circumstances, such as incubation duration and temperature, as well as bacterial extract type and application handling, might explain this phenomenon. An EDX examination revealed pure ZnO-NP phases and a prominent peak at 2.5 keV in the EDX spectrum, which indicated that the test sample contained pure zinc, according to the results. When the EDX spectrum was analyzed, it was shown that the pure ZnO-NPs with conspicuous peaks had been effectively created. Additional peaks in the spectrum, on the other hand, were discovered, indicating that bacterial biomolecules were involved in the production of nanoparticles. It was revealed that the EDX pattern of high purity ZnO-NPs and low purity ZnO-NPs is identical [33]. A similar study utilizing EDX analysis to assess the purity of ZnO-NPs was also carried out, with pure Zinc being used as a control. The presence of extra peaks in the spectrum shows that the sample was pure. Additionally, the size and crystallinity of the biosynthesized zinc oxide nanoparticles were measured using an X-ray diffraction (XRD) analysis of the samples. The XRD spectrum of ZnO-NPs indicated that they had a planar orientation and a crystalline structure. The primary XRD peaks were discovered at (100), (002), (101), (102), (110), (103), and (112) degrees, with 2 values of 21.7°, 26.11°, 33.16°, 35.11°, 39.61°, 48.88°, and 56.31°, respectively. The XRD peaks were discovered at (100), (002), (101), (102), (110), (103), and (112) degrees, respectively. The presence of several XRD reflection planes indicates that ZnO-NPs have a face-centered cubic (fcc) crystalline structure. In this study, an average crystal size of 48.22 nm was measured, which corresponded to the International Center of Diffraction Data card (JCPDS-36–1451), which indicated that a hexagonal crystal structure had been produced [29]. Four pathogenic bacteria strains, Haemophilus felis, Haemophilus suis, Haemophilus bizzozeronii, and Haemophilus salomonis, were examined in the presence of NPs at concentrations ranging from 5 mg/mL to 0.5 mg/mL. The results showed that all four harmful bacteria strains were susceptible to NPs. A bactericidal effect of the ZnO-NPs was shown at high concentrations, whereas a bacteriostatic effect was demonstrated at low quantities. A robust inhibitory zone of 116.21 ± 0.35, 12.94 ± 0.22, 19.53 ± 0.62, and 14.23 ± 0.72 was seen for Helicobacter salomonis, Helicobacter felis, Helicobacter bizzozeronii, and Helicobacter suis against a concentration of 5 mg/mL in all four Helicobacter species tested. As a result of their interaction with bacterial cell membranes, nanoparticles may cause damage by leaking internal components into the surrounding environment. Because of their small size, nanoparticles' antibacterial, antifungal, and other biological actions are largely reliant on their concentration; nanoparticles with small diameters are able to easily penetrate bacterial protective barriers and inflict damage [25]. The data presented here demonstrate that ZnO-NPs are a potent therapeutic agent for the reduction of inflammation in acute situations. To be eligible to receive the diclofenac (100 mg/kg), the group treated with the ZnO-NPs had 83.52 ± 0.18 percent less edema than the control group after 48 hours. ZnO-NP-treated mice showed 83.520.18% reduction of edema after 48 hours when compared to the control category, which received diclofenac (100 mg/kg). This was a significant improvement over previous findings. In addition to inhibiting histamine, serotonin, and prostaglandin, ZnO-NPs are known to possess anti-inflammatory properties that are expected to be substantial [34]. After 21 days, ZnO-NPs showed an 87.62 ± 0.112 percent reduction in edema. Several herbal remedies have been shown to lessen the severity of the illness in the rat adjuvant arthritis model, including echinacea. Antioxidant activity has also been linked to a decrease in the symptoms and inflammation associated with arthritis [34]. Using diabetic mice, this research demonstrated that the injection of zinc-based nanoparticles leads in large decreases in blood glucose levels, even when the animals are not yet fasting. We observed that ZnO-NPs significantly reduced glucose levels in diabetic mice that had been induced with STZ. The blood glucose levels of mice treated with ZnO-NPs and mice treated with the combination of ZnO-NPs and insulin were lowered by 36.89 percent and 74.38 percent, when compared to the control group, respectively. STZ enters the cell by a glucose transporter and causes DNA damage through alkylation as it travels through the cell [35]. The impact of ZnO-NPs on the structure of the kidneys of mice was investigated by the use of intraperitoneal injection. The diameter of the glomerular corpuscles (45.87 ± 0.23 mm), Bowmann's space (16.21 ± 0.53 mm), proximal (49.32 ± 0.52), and distil tubules (53.61 ± 0.82) were measured after 14 days of intraperitoneal injection of mice with 150 mg kg^−1^ ZnO-NPs. The proportion of hemolyzed ZnO-NPs demonstrated the cytotoxic nature of these newly generated ZnO-NPs at the highest concentrations; i.e., at 400 g/mL, the percentage was 4.54 ± 0.31, and at 12.5 g/mL, the percentage was 2.36 ± 0.24, in accordance with the findings reported by [36], which were identical. Our results support the biosafety of ZnO-NPs by revealing that the ZnO-NPs generated by the bacteria Paraclostridium benzoelyticum strain 5610 are stable and helpful in a variety of applications.

## 5. Conclusions

The formation of ZnO-NPs by ecofriendly and bioprinting with bacteria Paraclostridium benzoelyticum strain 5610 biomass was demonstrated in the current work. The ZnO-NPs were analyzed using various analytical methods, including UV, XRD, FTIR, SEM and EDX. In vitro and in vivo studies have demonstrated that this ZnO-NPs formulation possesses antibacterial, antidiabetic, and anti-inflammatory activities. ZnO-NPs showed vigorous antimicrobial and antidiabetic activity due to the findings, which might lead to the development of novel antibacterial and antidiabetic medications. However, this study looked at the molecular processes and physiological aspects of ZnO-NPs; further research is needed to understand the link between these mechanisms and the clinical consequences observed in mice. The introduction of Bioconstituents that are both active and have higher characteristics has boosted the biological uses of ZnO-NPs. As a result, the bacteria Paraclostridium benzoelyticum strain 5610 extract might be an effective alternative to traditional treatments.

## Figures and Tables

**Table 1:** ZnO-NPs have an antidiabetic impact in STZ-induced diabetic mice.

Groups	Dose	Blood glucose levels (mmol/L) fasting glucose levels	Glucose levels after 2 h	% of inhibition
Control distal water	1 mL/mice (oral)	19.46 ± 0.25	19.63 ± 0.29	—
NPs	14 mg/kg b.w. (oral)	19.28 ± 0.41	12.25 ± 0.19	48.63
NPs+insulin	7 mg/kg b.w.(oral) + 0.2 U/50 g (s.c.)	19.48 ± 0.26	14.63 ± 0.21	36.89
G-IV (standard: insulin)	0.4 U/50 g (s.c.)	19.57 ± 0.37	9.5.31 ± 0.48	55.71

**Table 2:** Hypoglycemic activity of ZnO-NPs.

Groups	Dose	Blood glucose levels (mmol/L) fasting glucose levels	Glucose levels after 2 h	% of inhibition
Control distal water	1 mL/mice (oral)	8.10 ± 0.23	6.85 ± 0.06	—
NPs, mild dose	8 mg/kg b.w. (oral)	8.62 ± 0.34	4.63 ± 0.14	49.94
NPs, moderate dose	14 mg/kg b.w. (oral)	7.38 ± 0.38	2.87 ± 0.28	74.38
Standard: glibenclamide	10 mg/kg b.w. (oral)	8.08 ± 0.27	3.37 ± 0.32	48.12

**Table 3:** Changes in the diameter of glomerular corpuscles and Bowmann's space distance in mice after 7 and 14 days of intraperitoneal injection of 150 mg kg^−1^ ZnO-NPs.

Treatments	Duration of injection (day)	Glomerular diameter (*μ*m)	Bowmann's space (*μ*m)
Control	7	56.21 ± 0.42	11.41 ± 0.06
150 mg/kg NP dose	7	56.62 ± 0.31	17.42 ± 0.15
Control	14	64.37 ± 0.42	9.42 ± 0.31
150 mg/kg NP dose	14	45.87 ± 0.23	16.21 ± 0.53

**Table 4:** Changes in the diameter of the proximal and distil tubules were detected after intraperitoneal administration of 150 mg kg^−1^ ZnO-NPs into mice for 7 and 14 days.

Treatments	Duration of injection (day)	Proximal tubule diameter (*μ*m)	Distil tubule diameter (*μ*m)
Control	7	38.62 ± 0.81	41.93 ± 0.18
150 mg/kg NP dose	7	46.72 ± 0.64	48.31 ± 0.32
Control	14	40.61 ± 0.36	41.89 ± 0.42
150 mg/kg NP dose	14	49.32 ± 0.52	53.61 ± 0.82

## Data Availability

All required data is present in this file.
